# A Metabolic Biofuel Cell: Conversion of Human Leukocyte Metabolic Activity to Electrical Currents

**DOI:** 10.1186/1754-1611-5-5

**Published:** 2011-05-10

**Authors:** Gusphyl A Justin, Yingze Zhang, X Tracy Cui, Charles W Bradberry, Mingui Sun, Robert J Sclabassi

**Affiliations:** 1 Department of Bioengineering, University of Pittsburgh, Pittsburgh, PA, USA; 2 Department of Medicine, School of Medicine, University of Pittsburgh, Pittsburgh, PA, USA; 3 Department of Neurological Surgery, University of Pittsburgh, Pittsburgh, PA, USA; 4 Department of Psychiatry, University of Pittsburgh, Pittsburgh, PA, USA; 5 Computational Diagnostics, Inc, Pittsburgh, PA, USA; 6 GJ is currently a National Research Council (NRC) Postdoctoral Fellow at the Center for Bio/Molecular Science and Engineering at the US Naval Research Laboratory, Washington, DC, USA

## Abstract

An investigation of the electrochemical activity of human white blood cells (WBC) for biofuel cell (BFC) applications is described. WBCs isolated from whole human blood were suspended in PBS and introduced into the anode compartment of a proton exchange membrane (PEM) fuel cell. The cathode compartment contained a 50 mM potassium ferricyanide solution. Average current densities between 0.9 and 1.6 μA cm^-2 ^and open circuit potentials (V_oc_) between 83 and 102 mV were obtained, which were both higher than control values. Cyclic voltammetry was used to investigate the electrochemical activity of the activated WBCs in an attempt to elucidate the mechanism of electron transfer between the cells and electrode. Voltammograms were obtained for the WBCs, including peripheral blood mononuclear cells (PBMCs - a lymphocyte-monocyte mixture isolated on a Ficoll gradient), a B lymphoblastoid cell line (BLCL), and two leukemia cell lines, namely K562 and Jurkat. An oxidation peak at about 363 mV vs. SCE for the PMA (phorbol ester) activated primary cells, with a notable absence of a reduction peak was observed. Oxidation peaks were not observed for the BLCL, K562 or Jurkat cell lines. HPLC confirmed the release of serotonin (5-HT) from the PMA activated primary cells. It is believed that serotonin, among other biochemical species released by the activated cells, contributes to the observed BFC currents.

## Background

Presently, there are few options for supplying power to implantable medical devices. The ultimate goal of this preliminary work is to develop an implantable biofuel cell device that may be used within the physiological environment for low-power implantable medical device applications (such as miniature biosensors) [1-3]. A biofuel cell (BFC) is an electrochemical or galvanic device that couples the oxidation of a biofuel (such as glucose) at the anode to the reduction of molecular oxygen to water at the cathode. Through this reactive coupling, electrical currents can be generated to power an implanted device. With the movement of electrons through the circuit from the anode to the cathode through the device, it is necessary to also have the simultaneous movement of positive charge between the two electrodes to satisfy the requirements of a closed circuit. This positive charge takes the form of protons that travel from the anode through an electrolyte to the cathode where water is the final byproduct.

Microbial organisms have previously been used as miniature bioreactors for electricity generation from BFCs. The microbes metabolize a substrate (such as glucose or acetate) and subsequently transfer high energy electrons to the anode of the BFC [4-14]. Electrons derived from these biofuels are subsequently transferred to the anode across the plasma membrane of the cells, while protons are also simultaneously released by the cells into the extracellular space. In another type of BFC - referred to here as an enzymatic biofuel cell (EnzBFC) for differentiation - specific enzymes are immobilized at the anode and cathode [15-21]. At the anode, glucose oxidase may be used to oxidize glucose to gluconolactone, while a laccase enzyme or bilirubin oxidase may be tethered to the cathode surface to reduce oxygen to water.

Electron transfer between cellular metabolic processes and electrodes has previously only been observed for microbes confined to the anode of a biofuel cell (BFC). Electron mediators, such as neutral red, have been employed to increase the efficiency of electron transfer between the microbes and the electrode surface [6]. Other microbes, such as Geobacter, have been shown to be capable of directly transferring electrons to an electrode without the aid of mediators and are often termed metal-reducing bacteria [11-13]. In this study, we investigate the feasibility of transducing the biochemical energy of white blood cells into electrical energy, essentially utilizing these eukaryotic cells as bioreactors at a biofuel cell anode.

## Methods

### A. Measurement of open circuit potential and current from BFC device

This study was approved by the Institutional Review Board (IRB) for Human Subject Research of the University of Pittsburgh. White blood cells (WBC) were isolated from 5 healthy adult human subjects using a red blood cell (RBC) lysis technique (Qiagen, Valencia, CA). Approximately 10 mL of anticoagulated peripheral blood was mixed with three volumes of RBC Lysis solution and incubated for 10 minutes at room temperature. WBC was recovered by centrifugation at 930 *g *for 10 minutes at 4°C. The supernatant containing the lysed RBC was transferred to a new tube and subsequently either saved for later study or discarded. The remaining WBC pellets were washed multiple times (at least thrice) in 1 × phosphate buffered saline (PBS) solution and resuspended in PBS to a final volume of 15 mL.

For specific isolation of peripheral blood mononuclear cells (PBMC), a Ficoll-Paque™ density gradient was used. Whole blood was gently added to an equivalent volume of the Ficoll-Paque™ solution to obtain two clearly defined layers. After centrifugation at 930 *g *for 20 minutes, four layers can be discerned - red blood cells at the bottom, followed by a larger volume of the Ficoll solution, then a thin layer of white blood cells - the PBMCs (consisting primarily of B and T lymphocytes and monocytes) - and finally a larger volume of blood plasma. The PBMCs were carefully recovered and washed twice in PBS (pH 7.4) by centrifugation at 800 *g *for 15 minutes and were finally resuspended in 15 mL PBS.

**Figure 1:**
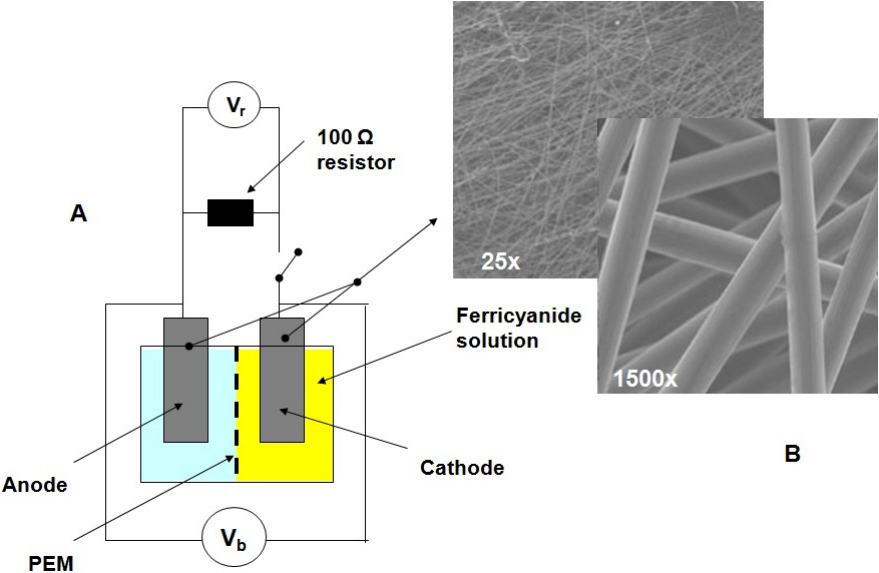
**(A) WBC biofuel cell experimental setup; (B) SEM of carbon fiber electrode**.

Multimeters were used to measure the open-circuit potential, V_oc_, as well as the potential across a 100 Ω resistor, V_r_, during current flow. Current, I (μA), and current density, J (μA cm^-2^), were found using Ohm's law. The surface area of the electrodes was taken as the *geometric *surface area and *not *the actual surface area. Woven carbon fiber electrodes (8 cm^2 ^geometric surface area and approx. 0.47 m^2 ^g^-1 ^specific surface area) were used for both the anode and cathode (Figure 1). The cell suspensions were confined to the anode compartment, while the cathode compartment contained a 50 mM potassium ferricyanide (K_3_[Fe(CN)_6_]) solution in 1 × PBS (pH 7.4). Anode and cathode were separated by a proton exchange membrane (Nafion-117, Sigma-Aldrich Co, St. Louis, MO). WBC activation was achieved with 5 ng mL^-1 ^phorbol-12-myristate-13-acetate (PMA) and 500 ng mL^-1 ^calcium ionomycin (final concentrations in solution, Sigma-Aldrich, St Louis, MO). PMA activates the protein kinase C (PKC) pathway which is involved in numerous signal transduction cascades leading to events such as respiratory burst in macrophages and aggregation of blood platelets. The components used to construct the BFC, including the walls, chamber and carbon fiber electrodes, were purchased from the National Center of Biotechnology Education, University of Reading, UK (Figure 1).

### B. Cyclic voltammetry

Cyclic voltammetry was performed using the Gamry Potentiostat, FAS2/Femtostat under control of the Gamry Framework Software from Gamry Instruments (Warminster, PA). A three-electrode setup was used, where carbon fiber and platinum served as the working and counter electrodes, respectively, while a saturated calomel electrode (SCE) served as the reference. PMA (5 ng mL^-1^) and calcium ionomycin (500 ng mL^-1^) were used to activate the white blood cells. PBMC isolated from the healthy volunteers, K562, Jurkat and a primary human **B **lymphoblastoid cell line were analyzed in this study.

Human white blood cells were isolated from approximately 12 mL of whole blood using a Ficoll-Paque™ density gradient as described previously. Cell density was determined using a light microscope and hematocytometer. K562, BLCL and Jurkat cell lines were also cultured over a period of one week in RPMI with 10% fetal bovine serum (FBS). The cells were isolated from the culture medium by centrifugation, washed twice with PBS and finally resuspended in 1 × PBS. Cell density was determined as described above.

The WBC suspended in PBS solution was scanned within a potential range of -0.5 V to 1.2 V vs. SCE at a scan rate of 100 mV s^-1^. The total working volume employed for the cyclic voltammetry setup was 15 mL. The white blood cells were activated by addition of 1 μL each of phorbol-12-myristate-13-acetate (PMA) and ionomycin per 1 mL of the total working volume to achieve final concentrations of 5 ng mL^-1 ^and 500 ng mL^-1^, respectively. The effect of variable glucose concentrations was investigated.

**Figure 2:**
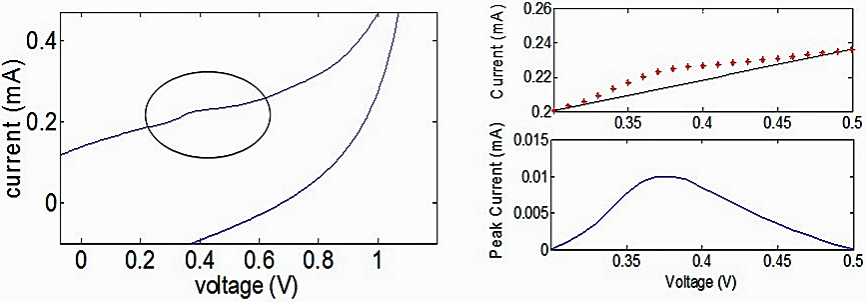
**Current-potential (i-V) curve acquired from the cyclic voltammetry of activated human WBCs suspended in PBS**. An oxidation peak is observed at ca. 400 mV.

The peak potentials and currents associated with the observed oxidation peaks were determined using MATLAB^® ^software. Specifically, any oxidation peaks present in the cyclic voltammograms were identified and the electric potential range for the region of interest corresponding to the oxidation peak defined (Figure 2). Upper and lower boundaries corresponding to the region of interest were determined by visual inspection to identify the highest and lowest electric potentials (voltages) corresponding to the width of the identified oxidation peak. Linear regression and interpolation were then used to fit a baseline bounded by the two points corresponding to these upper and lower potentials. The interpolated current values for the baseline were subtracted from the original current values of the cyclic voltammogram within the region of interest corresponding to the oxidation peak. As a result, adjusted values for the peak current were obtained.

In vitro microdialysis [22] was used in these experiments to extract the released biochemical content from the cell suspension, while high performance liquid chromatography (HPLC) with electrochemical detection was subsequently used to validate whether or not serotonin was released by the cells, as well as to quantify the amount of serotonin released upon cell activation. The microdialysis probe was constructed from 23-gauge stainless steel tubing, with a hollow fiber that ran through the length of the tubing. A small length of the hollow fiber (approx 1.5 - 2.0 mm) was allowed to protrude from the end of the stainless steel tube and remained in contact with the surrounding solution. Within the hollow fiber was vitreous silicate tubing. This silicate tubing served as an inlet section for the perfusing buffer, while the outlet section was housed in the 23-G steel tubing. Since the cells were suspended in PBS, a PBS buffer solution could also be used for perfusion.

White blood cells were isolated as described previously. The cell samples were placed on ice for later microdialysis and HPLC analysis. K562, BLCL and Jurkat cell lines were also analyzed. 1.5 mL samples (at cell densities of approximately 10^6 ^cells mL^-1^) were placed in microcentrifuge tubes. The hollow fiber to be used for microdialysis was allowed to equilibrate in fresh distilled water (dH_2_O) for a period of 30 minutes to one hour. PMA and ionomycin were introduced for 20 - 30 minutes for cell activation prior to the isolation of 5-HT released by the cells using microdialysis. Three samples were collected from microdialysis of the cell suspensions and controls (perfusion rate of 5 μL min^-1 ^for 2 minutes) in autosampler tubes. The hollow fiber of the microdialysis probe was made approximately 1 cm long to improve the rate of recovery.

For washing, the hollow fiber was placed in fresh distilled water (dH_2_O) and allowed to dialyze at a rate of 20 μL min^-1 ^for 2 minutes. It was then placed in fresh dH_2_O for 3 minutes between samples to wash out any residual 5-HT. 8.2 μL sample volumes were injected into the HPLC by a FAMOS autosampler. Detection of any 5-HT was determined electrochemically using an Antec-Leyden Intro amperometric detector (Zoeterwoude, The Netherlands).

## Results

### A. Measurement of Open Circuit Potential and Current from BFC device

**Figure 3:**
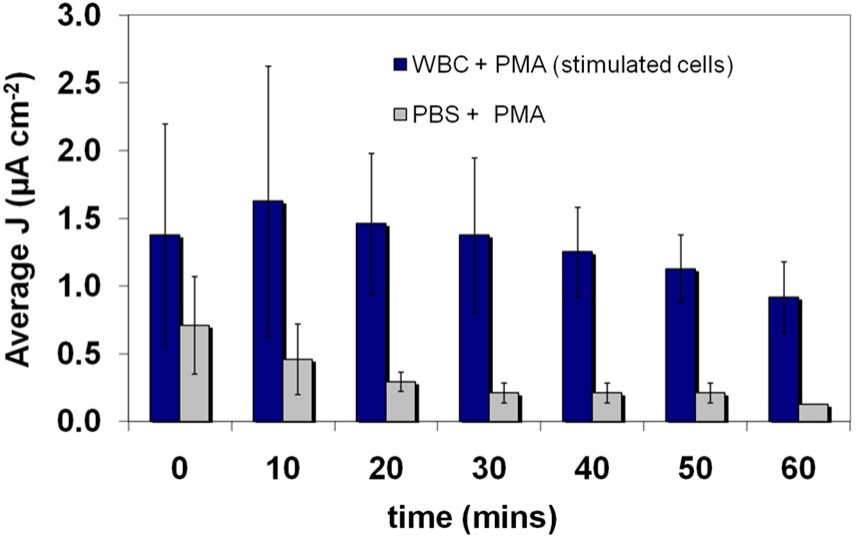
**Currents produced from the PEM fuel cell following introduction of activated WBCs and subsequent replacement of the WBC suspension with PBS (n = 3)**. A significant decrease in the current output occurs, indicating that the WBC suspension is the primary contributor to the observed currents (p < 0.05).

Average current densities between 0.9 and 1.6 μA cm^-2 ^(n = 3) and average open circuit potentials between 83 and 102 mV (n = 3) were measured when a proton exchange membrane (PEM) fuel cell (PEMFC) containing WBCs isolated by RBC lysis was placed in series with a 100 Ω resistor (Figure 3). There was significant variation in the observed currents, with standard deviations reaching as high +/- 61%. Over time; however the standard deviations of the current density values across the three WBC samples declined appreciably suggesting that the system was gradually achieving stability. On replacing the WBC suspension with a solution of the activating agents (PMA and ionomycin) dissolved in PBS, a decrease in the current density was observed compared to the WBC suspension. In the case of this control, a similar phenomenon was observed where the standard deviation obtained from the three separate experiments decreased over time. Again, this was attributed to the attainment of stability within the system. The observation of small currents for the control is an indication that the PMA and/or ionomycin have some electrochemical activity. However, the results illustrated in Figure 3 suggest that the white blood cells are also transferring electrons to the electrode surface, contributing to the higher magnitude currents. The production of metabolic products by the cells can be implicated, just as bacterial cells in microbial fuel cells have been shown to produce soluble metabolic products that are oxidized at the fuel cell anode. A one-tailed student's t-test of the experimental and control data reveal a p value less than 0.05 (p = 1.1 × 10^-6^) indicating that the current densities recorded are significantly different.

**Figure 4:**
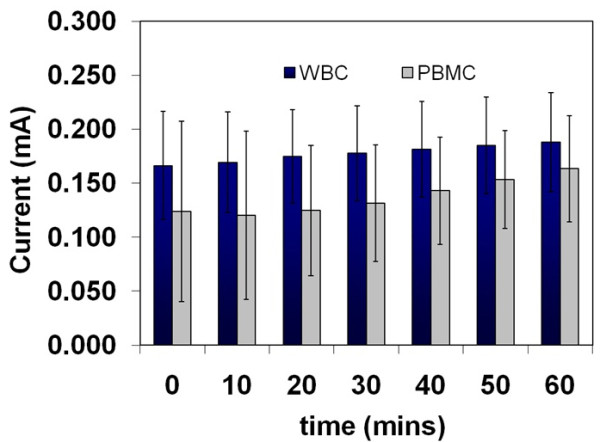
**Comparison of currents obtained from WBCs and PBMCs**.

**Figure 5:**
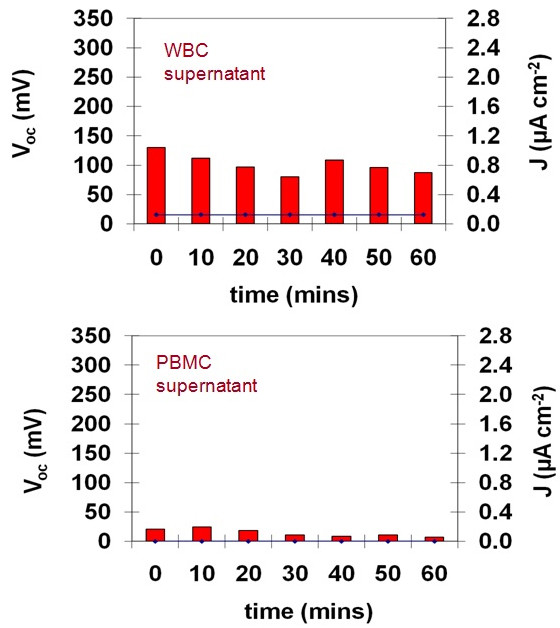
**Comparison of open circuit potentials (bars) and currents (lines) obtained from the supernatant (PBS used to wash the cells during the initial Ficoll isolation procedure) for both the WBCs (top) and PBMCs (bottom)**. Relatively small currents to no currents were observed for the PEM fuel cell apparatus.

The current output from PBMCs (isolated on a Ficoll-Paque ™ gradient) within the same experimental setup was also measured. The average current from the PBMC was slightly lower than that recorded for the general WBC population - which contains several other cell types, including neutrophils in addition to the PBMCs (Figure 4). A PEMFC containing PBS only (without activating agents) at the anode did not produce any observable currents or open circuit potentials (not shown). Introducing samples of the PBS supernatant, used in the initial washing of both the WBC and PBMC samples subsequent to isolation, into the anode compartment of the PEMFC produced very small to no currents (Figure 5). This further supports the theory that the cells, and not residual components from the cell isolation procedure, are primarily responsible for the observed currents. Frequent measurements of pH made during the course of all experiments also did not reveal any significant changes across all anode solution samples (pH maintained at 7.4 regardless of cell density, cell activation or due to the presence of the activating agents).

These results imply that within the general WBC population, certain cells or the molecules released by certain cells are electrochemically active at the electrode surface. These can be attributable to the oxidation of electrochemically active species that are either released by the cells into the extracellular environment (such as serotonin) or by redox active species that are present in the cell membrane (such as flavohemoproteins of the NADPH oxidase complex, coenzyme A). In order to be able to produce a current from the BFC, protons must also be released by the cells into the extracellular space. The increase in proton concentration in the anode compartment is important to create a diffusion gradient, whereby the protons can traverse the PEM to react with molecular oxygen to form water at the cathode. Placing either PBS solution or deionized water in both the anode and cathode compartments did not produce any detectable currents and were associated with open circuit potentials that were less than 20 mV.

### B. Cyclic Voltammetry

Cyclic voltammetry (CV) was utilized to investigate the electrochemical activity of the isolated human white blood cells. Cyclic voltammograms acquired from PMA (5 ng mL^-1^) and ionomycin (500 ng mL^-1^) activated human WBCs and PBMCs revealed oxidation peaks at about 360 mV vs. SCE and peak currents up to 12 μA for cell densities of 10^6 ^cells mL^-1 ^(Figure 2). Oxidation peaks were not observed for any other cell type, i.e. K562, Jurkat and BLCL cells. There was a noticeable absence of a reduction peak in the voltammograms, which normally implies that irreversible oxidation of the species has occurred. Peaks were neither observed in the absence of cells nor before activation of the cells with PMA. The results of our experiments suggest that the activated lymphocyte-monocyte mixture comprising the PBMCs releases redox active species into the extracellular environment upon activation. Based on studies performed by previous research groups (Table 1), it is likely that the redox species responsible for the 360 mV oxidation peak is 5-HT [23-26], although one paper cited membrane bound proteins as the source of the oxidation peak [27]. It has been suggested that 5-HT is released from certain human leukocytes during inflammatory responses, particularly in response to allergens during allergic reaction. In these studies, the oxidation peak for 5-HT was normally detected at around 330 mV vs. SCE, which is a slightly lower value than what we have detected (360 mV), although the difference was not significant. The absence of a reduction peak was often reported in these studies, and has been attributed to the irreversible oxidation of serotonin (5-HT) to 5-hydroxyindoleacetic acid (5-HIAA) [26].

**Table 1:** Cyclic voltammetry studies of white blood cells.

Reference	Cells	Working electrode	E_p_	I_p_	Electroactive species
Nakamura et al. (1991) [23]	Human leucocytes	Basal plane pyrolytic graphite	0.33 V and 0.68 V (leucocytes) and 0.68 V (erythrocytes)	0.36-0.58 μA per 10^6 ^cells for E_p _= 0.33 V	Serotonin (0.29-0.34 V) CoA (0.65-0.74 V for microorganisms)
Matsunaga et al. (1989) [24]	Rat basophilic leukemia cells (RBL-1) and mouse lymphocytes	Basal plane pyrolytic graphite	0.34 V and 0.68 V (RBL-1) 0.65 V (mouse lymphocytes)	0.76 μA per 10^5 ^cells for E_p _= 0.34 V	Serotonin (0.29-0.34 V) CoA (0.65-0.74 V for microorganisms)
Ci et al. (1998) [27]	White rabbit leukocytes and erythrocytes	Graphite	0.32 V (leukocytes) 0.73 V (erythrocytes)	1 μA per 10^5 ^cells for E_p _= 0.32 V	Membrane-bound proteins?

All measurements were made in PBS with a saturated calomel electrode (SCE) as the reference electrode.

The oxidation peaks obtained from the cell suspensions were barely discernable against the background currents, as the total area bounded by each CV curve was very large by comparison to the area bounded by the oxidation peaks within the 350 mV to 400 mV range (Figure 2). The large background currents are a direct consequence of two factors, namely the scan rate (set at 100 mV s^-1^) and the large surface area of the carbon fiber electrodes (PRF Composite Materials, Dorset, England). Greater surface area of the electrode leads to greater capacitance. The relationship between the scan rate, capacitance and voltammetric current can be expressed by the following equation:(1)

where *i *is current, *v *is the scan rate, *C_d _*is the capacitance of the electrode, *t *is time and *R_s _*is solution resistance [28]. The peak current for an irreversible reaction is described by the following equation:(2)

where *i_p _*is peak current, *α *is the electron transfer coefficient, *n *is the number of electrons transferred, *A *is surface area, *D_o _*is the diffusion coefficient, C_o_* is the concentration of the species in the bulk solution and *v *is the scan rate [28].

**Figure 6:**
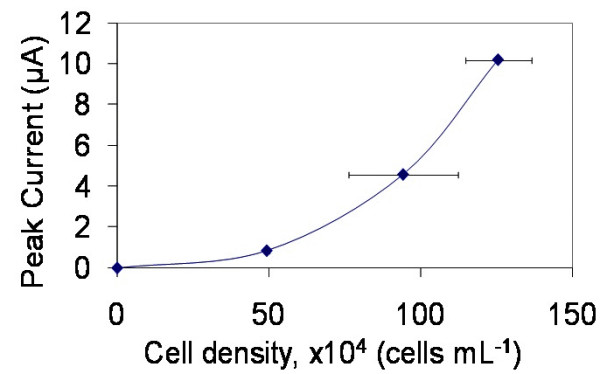
**Variation in the amplitude of the oxidation peak current is plotted with PBMC density**. Increasing the cell density is associated with an apparent non-linear increase in the oxidation peak.

The 30 mV difference between the peak potentials reported in the literature (330 mV) and our own (360 mV) is not interpreted to be meaningful. 30 mV may not be significant, since scan conditions and electrode surface features can significantly impact peak potential. Figure 6 shows that the magnitude of the oxidation peak current increases with cell density, further verifying the relationship between the cells and the electrochemical activity. The relationship between cell density and peak current in Figure 6 appears to be nonlinear. However, this may only be an apparent nonlinearity as variability in how strongly cells from different subjects respond to the PMA activation agent can occur. As a consequence, this would affect how much serotonin is ultimately produced.

It is well known that several types of cells contain protein complexes in their cell membranes that function as electron transport chains. The NADPH oxidase complex, for example, comprises structures within the plasma membrane that upon association in an activated state transfer electrons between the pentose phosphate pathway of glucose metabolism and extracellular oxygen. Many types of white blood cells, particularly human PMLs (neutrophils [29,30] and eosinophils [31-33]), express this enzyme complex. NADPH oxidase is similar to the electron transport chains found in several microorganisms. Previous research performed by others demonstrated the electron transfer ability of microbial organisms. It is known that these microorganisms can transfer electrons to electrodes by various mechanisms, including: i) the use of artificial electron mediators (electronophores) such as neutral red; ii) using mediators produced by the microorganisms themselves; iii) direct electron transfer across the bacterial cell membrane [6]. Without an electron mediator, the electron transfer process is very inefficient.

### C. Microdialysis and HPLC

**Figure 7:**
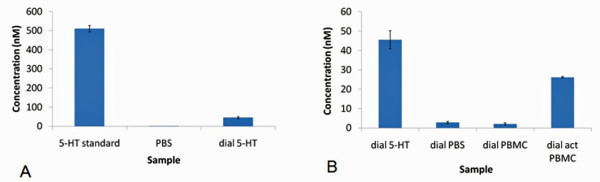
**Release of serotonin from activated WBCs and more specifically, PBMCs**. Following the microdialysis ("dial") of 5-HT (500 nM), PBS, PBMCs and activated PBMCs, 5-HT was determined using HPLC. A recovery estimate of 9% (+/- 1%) was determined for the microdialysis probe (as calculated from 5-HT standard and dial 5-HT in A). Based on this estimate, a 5-HT concentration of approximately 291 nM (+/- 34 nM) was calculated to be associated with the activation of PBMCs at a cell density of 10^6 ^cells mL^-1 ^(B). 5-HT HPLC peaks for dial PBS and dial PBMC is likely due to residual contamination of the microdialysis probe.

The HPLC results confirmed that serotonin was in fact released from PMA/ionomycin activated white blood cells, specifically PBMCs (Figure 7). A 5-HT concentration of approximately 291 nM (+/- 34 nM) was calculated to be associated with the activation of PBMCs at a cell density of 10^6 ^cells mL^-1^. In contrast, no 5-HT was detected from the K562, BLCL or Jurkat cells. The results of this experiment indicate that increased extracellular concentrations of serotonin, from the activated PBMCs, are directly associated with the oxidation peaks observed in the *i-V *curves obtained using cyclic voltammetry. The fact that none of the cell lines were associated with oxidation peaks suggests that these cells do not release serotonin. This was verified by the absence of corresponding serotonin peaks in the HPLC chromatograms of these cells (not shown). These experiments highlight that important differences do exist between isolated human white blood cells and their corresponding cell lines cultured in vitro.

## Discussion

Electron transport, pervasive in the plasma membranes of microbes, is generally not observed across the extracellular membranes of eukaryotic cells. The presence of an electron transport chain in the extracellular membranes of microbes provides a likelihood of electron tunneling occurring between the membrane-bound species and the electrode surface. In eukaryotic cells, electron transport is a process that is normally relegated to the membranes of intracellular mitochondria. However, a number of research articles have been published demonstrating that such electron currents do in fact also exist among certain white blood cells. Schrenzel et al. (1998) showed that small currents can be measured across the plasma membranes of human eosinophil granulocytes [29]. The experiments were performed based on the hypothesis that the membrane associated enzyme NADPH oxidase, found in blood phagocytes (e.g. neutrophils and eosinophils), generates superoxide through electron transfer from NADPH to extracellular oxygen. The magnitudes of the currents recorded across the extracellular membranes were on the order of 10 to 20 pA per cell in these studies.

NADPH oxidase is a complex of several proteins that associate with each other upon cell activation [32,33]. Activation in vitro is normally achieved by application of a phorbol ester, phorbol-12-myristate-13-acetate (PMA) [29,30,33]. Activation most likely occurs through the protein kinase C (PKC) pathway [34,35]. The enzyme complex and its analogs have also increasingly been found in a variety of different cell types including microglia [36], vascular smooth muscle [37], hematopoietic stem cells [38], and endothelial cells [39]. The presence of an electron transport chain in the plasma membranes of white blood cells means that it may be possible to "hijack" these high energy electrons in order to divert them to a nearby electrode. Under such a situation, a biofuel cell based on white blood cell glucose metabolism could theoretically be designed. The electrical interactions between the cells and the electrode surface become important for such a direct electron transfer process to take place.

The idea of using electrons derived from NADPH oxidase to power a biofuel cell was first proposed by Justin and colleagues [1-3,40-45] and then more recently by Sakai and colleagues [46]. In the studies performed by Justin and colleagues, including this one, human white blood cells were isolated directly from whole human blood. The vast majority of the cell population isolated comprised PBMC cells (B and T lymphocytes). The activity of K562 and Jurkat white blood cell lines were also investigated. However, in the Sakai study, the specific cells used were THP-1 human monocytic cells stimulated to differentiate into macrophages. The Sakai study demonstrated that the current output from a BFC incorporating macrophages at the anode could be disrupted by application of NADPH oxidase inhibitors such as diphenylene iodinium (DPI). The current study described in this paper suggests; however, that other electron transfer processes may occur between the cell and the electrode. As demonstrated in this work, serotonin released from activated white blood cells is a likely mediator for electron transfer between the cells and the electrode. Despite confirmation of this by employing electrochemical techniques such as cyclic voltammetry and HPLC for validation, it is difficult to dispute the possibility that NADPH oxidase is also active. One of the reasons that our studies did not reveal CV redox peaks due to NADPH oxidase may have been due to NADPH oxidase not being as active among PBMCs compared to macrophages such as neutrophils and eosinophils. Only very low levels of macrophages would normally be isolated from healthy individuals, as was the case in our study - a necessary requirement for Institutional Review Board (IRB) compliance. Therefore, the effect of NADPH oxidase activity specifically on the biofuel cell currents would not have been as predominant as culturing differentiated macrophages onto electrodes as performed by Sakai and colleagues. It is surprising that in the work by Sakai and colleagues that a larger background current associated with serotonin was not observed. This suggests that serotonin may not be produced by the differentiated monocytes, unlike the phenomenon of release demonstrated by the cells used in our studies. Vastly different mechanisms of electron transfer for the specific white blood cells used in the two studies are very likely.

The idea of harvesting electrons from whole cells is not a new one. Previous studies performed by other researchers on microbial fuel cells suggested that under normal conditions, it is very difficult to achieve direct electron transfer between the cell membrane components of the microbes and an interfacing electrode [14]. An important reason for this is the fact that the redox active groups are generally embedded within integral membrane proteins, resulting in a significant energy barrier that increases with increased distance between electron donor and receptor pairs. Electron tunneling between the membrane components and the electrode surface is further discouraged because of the thermodynamically unfavorable conditions surrounding the movement of free electrons through an aqueous solution. In order to overcome this challenge, many microbial fuel cell researchers employed electron mediators that were capable of shuttling electrons between the membrane-bound redox active components of the microbes and the electrode. Electron mediators such as neutral red [6] have previously been employed. The use of these electron mediators significantly increased the efficiency in electron transfer and thus the current output of the microbial fuel cells.

A future goal in continuing the work described in this paper would be to explore means of enhancing the electron transfer efficiency of human leukocytes for enhancing current and power densities in implantable biofuel cell applications.

## Conclusions

In this study, we proposed the development of a novel biological fuel cell that can utilize the body's own resources to generate electricity, through specific electrochemical interactions between cells and electrodes in close proximity. The motivation of this study is to develop a BFC that can be used to power implantable medical devices, including micro- and nano- biosensors for either therapeutic or physiological monitoring purposes. The study seeks to demonstrate that electron transfer between human white blood cells and an interfacing electrode can occur through any or all of three possible mechanisms: 1) direct electron transfer through membrane bound redox species (such as the flavocytochrome of NADPH oxidase); 2) indirect electron transfer through exocytosed non-metabolic biochemical species (e.g. serotonin); and 3) indirect electron transfer through exocytosed metabolically relevant biochemical species. Our results indicate that activated white blood cells can generate small electrical currents when introduced into the anode compartment of a proton exchange membrane fuel cell, with ferricyanide in the cathode compartment. Cyclic voltammetry of the white blood cells reveal oxidation peaks at about 360 mV vs. SCE. Peaks at this potential have been attributed to serotonin release. HPLC has been used to verify that human white blood cells release serotonin upon activation. Various white blood cell lines do not release serotonin, indicating that the isolated cells may uptake serotonin from the blood stream rather than metabolize it themselves.

## Competing interests

The authors declare that they have no competing interests.

## Authors' contributions

GJ performed the BFC current and potential measurements, electrochemical measurements and microdialysis and HPLC as well as drafted the manuscript. YZ was responsible for culturing the cell lines. XC helped in the design and implementation of the electrochemical experiments. CB helped in the design and implementation of the microdialysis and HPLC experiments. MS and RS conceived of the study and participated in the design and coordination of the overall study. All authors helped to draft the manuscript and have read and approved the final manuscript.
